# Virulent PB1-F2 residues: effects on fitness of H1N1 influenza A virus in mice and changes during evolution of human influenza A viruses

**DOI:** 10.1038/s41598-018-25707-y

**Published:** 2018-05-10

**Authors:** Irina V. Alymova, Jonathan A. McCullers, Ram P. Kamal, Peter Vogel, Amanda M. Green, Shane Gansebom, Ian A. York

**Affiliations:** 1 Influenza Division, National Center for Immunization & Respiratory Diseases, Centers for Disease Control & Prevention, Atlanta, GA USA; 2 Department of Pediatrics, University of Tennessee Health Sciences Center, Memphis, TN USA; 3 Departments of Infectious Diseases, St. Jude Children’s Research Hospital, Memphis, TN USA; 4 Departments of Pathology, St. Jude Children’s Research Hospital, Memphis, TN USA; 5 Battelle Memorial Institute, Atlanta, GA USA; 6 Present Address: Department of Graduate Medical Education, University of Tennessee Health Sciences Center, Memphis, TN USA; 7 Influenza Division, National Center for Immunization & Respiratory Diseases, Centers for Disease Control & Prevention, Atlanta, GA USA; 8 CNI Advantage, LLC, Norman, OK USA

## Abstract

Specific residues of influenza A virus (IAV) PB1-F2 proteins may enhance inflammation or cytotoxicity. In a series of studies, we evaluated the function of these virulence-associated residues in the context of different IAV subtypes in mice. Here, we demonstrate that, as with the previously assessed pandemic 1968 (H3N2) IAV, PB1-F2 inflammatory residues increase the virulence of H1N1 IAV, suggesting that this effect might be a universal feature. Combining both inflammatory and cytotoxic residues in PB1-F2 enhanced virulence further, compared to either motif alone. Residues from these virulent motifs have been present in natural isolates from human seasonal IAV of all subtypes, but there has been a trend toward a gradual reduction in the number of virulent residues over time. However, human IAV of swine and avian origin tend to have more virulent residues than do the human-adapted seasonal strains, raising the possibility that donation of PB1 segments from these zoonotic viruses may increase the severity of some seasonal human strains. Our data suggest the value of surveillance of virulent residues in both human and animal IAV to predict the severity of influenza season.

## Introduction

Influenza A viruses (IAV) cause disease in infected hosts through multiple mechanisms^1,2^. One such mechanism is promotion of a secondary bacterial pneumonia, a leading cause of death during IAV pandemics and severe epidemics^3–7^. Among the virus-encoded contributors to bacterial co-infection is the PB1-F2 protein encoded in an alternate open reading frame (ORF) of the PB1 segment of most human and zoonotic IAVs^8^. As a full length protein, PB1-F2 contains 87–90 amino acids; however, a number of IAV possess a truncated PB1-F2 (e.g. 11 or 57 residues in length) due to the presence of one or more premature stop codons^9–11^, or lack an initiating ATG^8^. Full-length PB1-F2 is believed to have impacted the severity and the ability to promote secondary bacterial pneumonia of the 1918(H1N1), 1957(H2N2) and 1968(H3N2) pandemic viruses, as well as H5N1 and H7N9 highly pathogenic avian influenza viruses (HPAIV)^12–17^.

PB1-F2 can increase cell death, potentiate inflammatory responses, impair cellular innate immunity, and upregulate viral polymerase activity^16,18–35^. Naturally-occurring variations in PB1-F2 amino acid sequence have been linked to its virulence^15^. We recently demonstrated the ability of a specific set of amino acid residues (L62, R75, R79, and L82) naturally present within the PB1-F2 C-terminus of pandemic 1968(H3N2) IAV to increase inflammation and promote secondary bacterial pneumonia in mice^16^; we refer to this set of amino acids as an “inflammatory motif”. Recent studies by McAuley *et al*.^36^ and Pinar *et al*.^17^ indicate that enhancement of inflammation by this motif might involve activation of the NLRP3 inflammasome, a mutliprotein oligomer responsible for initiation of inflammatory processes.

We also recently identified other C-terminal PB1-F2 residues (I68, L69, and V70) that promote development of secondary bacterial pneumonia in a mouse model of H1N1 infection. These residues lead to enhanced cytotoxicity^31^; hence we refer to this as a “cytotoxic motif”. The I68, L69, and V70 residues (along with F71) have been shown to increase the stability of the PB1-F2 protein^37^.

In addition to pandemic 1968(H3N2), the other major influenza pandemic viruses of the 20^th^ century (1918(H1N1) and 1957(H2N2)) also included a PB1 segment expressing a full-length PB1-F2 protein with each of the four inflammatory residues. The presence of inflammatory residues in PB1-F2 has been proposed to be among the reasons for the high virulence and excess mortality of these pandemic IAVs^16^. However, the impact on virulence of the inflammatory residues on IAV other than H3N2 has not been examined. This exploration is important, because the effects of PB1-F2 greatly differ between virus strains^15,27,38–40^. These differences may be partially explained by the fact that viruses containing full-length PB1-F2 ORF may express several different variants of the protein differing in their length^8^ and/or expression levels^41^ (which are regulated at the translational level by sequences within the PB1 gene segment)^42^, as well as by possible interactions with other viral proteins. Thus, the simple presence of residues within PB1-F2 that have been identified as virulent in one strain of IAV, may not guarantee that such an effect occurs in other strains.

Among pandemic viruses, only 1918(H1N1) had all four inflammatory residues (L62, R75, R79, and L82), but only had one cytotoxic residue, V70^31^. Other early seasonal H1N1 IAV viruses, such as A/Puerto Rico/8/1934 (PR8) and Wilson Smith Neurotropic (A/WSN/1933)^31^, have a combination of all four inflammatory residues and three cytotoxic residues (I68, L69, and V70). Presumably the co-existence of both types of residues could enhance PB1-F2 virulence; however, this has not been studied, and it remains possible that the effects are not additive, or might even be antagonistic.

While the presence of virulent PB1-F2 residues was demonstrated in pandemic 1918, 1957, and 1968 IAVs, their prevalence in human seasonal strains has not been evaluated. The presence of virulent PB1-F2 protein might be among the reasons for the severity of some seasonal IAV cases. Since inflammatory and cytotoxic residues are widespread within the PB1-F2 genes in animal (especially avian) IAV reservoirs^43,44^, this possibility raises the concern that introduction of the PB1 genomic segment from an animal IAV strain (either through reassortment or zoonotic infection) could significantly increase the virulence of seasonal human IAV.

To fill the gaps in our understanding of the biology of PB1-F2 virulent residues, here we analyze the nature and frequency of both inflammatory and cytotoxic residues in natural human IAVs to understand their potential for aggravation of seasonal influenza. We also determine the effect of inflammatory residues on virulence of the H1N1 IAV subtype, in order to evaluate the commonality of the phenomenon, and examine the combined effect of inflammatory and cytotoxic motifs. Because the laboratory-adapted PR8(H1N1) PB1-F2 contains the full set of both virulent residues and is an early representative of the H1N1 human subtype, we chose it as a model virus for our study.

## Results

### Prevalence of virulent residues in human influenza A virus PB1-F2

To understand the potential of zoonotic viruses for donating virulence-associated PB1-F2 to and distribution of these residues in human population, we assessed all PB1 segment sequences of IAVs isolated from human infections present in influenza databases on November 25^th^, 2017 (Table 1 and Supplementary Table 1), and found 28,688 of those covering the entire PB1-F2 ORF. These included not only pandemic and seasonal human viruses, but also viruses of swine or avian origin that caused zoonotic infections but that led to no, or very limited, human to human transmission. From 60.0% (swine-origin H1N2) to 100.0% (human seasonal H1N2) of the isolates from most of these strains had PB1-F2 of at least 62 residues, and were therefore capable of including at least L62. The exceptions were H1N1 human seasonal and pandemic 2009 (H1N1pdm2009) IAVs. Only 3.3% of human seasonal H1N1 isolates expressed PB1-F2 of 62 amino acid or longer. All of the H1N1 isolates with PB1-F2 longer than 62 residues predated 2010, when the seasonal strain was replaced by H1N1pdm2009 viruses, which have multiple stop codons that truncate PB1-F2^45^. Thus, none of the H1N1pdm2009 isolates expressed PB1-F2 that was long enough to contain virulent residues.

**Table 1:** Frequency of virulent residues* in PB1-F2 proteins of influenza A viruses isolated from humans.

Virus subtype	No. of assessed PB1 sequences	PB1 sequences coding for the region of virulent PB1-F2 residues^a^		Region of virulent PB1-F2 residues with virulent sequences		Virulent sequences with combination of inflammatory and cytotoxic residues		% of virulent residues in virulent PB1-F2						
		No.	%	No.	%	No.	%	1	2	3	4	5	6	7
Human pdm2009 H1N1	11,676	0	0.0	0	0.0	0	0.0	0.0	0.0	0.0	0.0	0.0	0.0	0.0
Human seasonal H1N1	1,535	49	3.2	49	100.0	24	49.0	12.2	34.7	4.1	4.1	4.1	16.3	24.5
Human pdm1918 H1N1	1	1	100.0	1	100.0	1	100.0	0.0	0.0	0.0	0.0	100.0	0.0	0.0
Swine origin H1N1	19	15	78.9	15	100.0	4	26.7	13.3	66.7	13.3	6.7	0.0	0.0	0.0
Human seasonal H1N2	30	30	100.0	1	3.3	0	0.0	100.0	0.0	0.0	0.0	0.0	0.0	0.0
Swine origin H1N2	10	6	60.0	6	100.0	1	16.7	0	83.3	16.7	0.0	0.0	0.0	0.0
Human seasonal H2N2	61	60	98.4	60	100.0	0	0.0	0	0	5.0	95.0	0.0	0.0	0.0
Human pdm1957 H2N2	17	17	100.0	17.0	100.0	0	0.0	0.0	0.0	5.8	94.2	0.0	0.0	0.0
Human seasonal H3N2	14,408	13,320	92.4	12,379	92.9	868	7.0	90.6	8.5	0.6	0.3	0.0	0.0	0.0
Human pdm1968 H3N2	17	17	100.0	17	100.0	0	0.0	0.0	0.0	0.0	100.0	0.0	0.0	0.0
Swine origin H3N2	146	145	99.3	145	100.0	2	1.4	1.4	93.8	3.4	1.4	0.0	0.0	0.0
Avian origin H5NX^b^	306	287	93.7	285	99.3	5	1.8	0.4	0.0	22.0	75.8	1.8	0.0	0.0
Avian origin H7NX^c^	643	569	88.5	560	98.4	116	20.7	4.6	1.1	7.2	66.4	20.7	0.0	0.0
Total	28,688	14,516		13,535		261								

^*^Inflammatory (L62, R75, R79, and L82) and cytotoxic (I68, L69, and V70) residues (according to PB1-F2 amino acid numbering).

^a^Residues 62–82 (at least 62 residues in length); bH5N1 and H5N6; cH7N2, H7N3, H7N7, and H7N9.

From 92.9% to 100% of human IAV isolates of all subtypes that were at least 62 residues long contained at least one virulent residue, except human seasonal H1N2 (a short-lived strain that circulated mainly in 2002–2003), in which 96.7% of viruses contained no virulent residues. Both inflammatory and cytotoxic residues, in various combinations, were observed in 1.8% to 50.0% of those PB1-F2 proteins except the now-extinct seasonal H1N2 and H2N2 strains.

**Figure 1:**
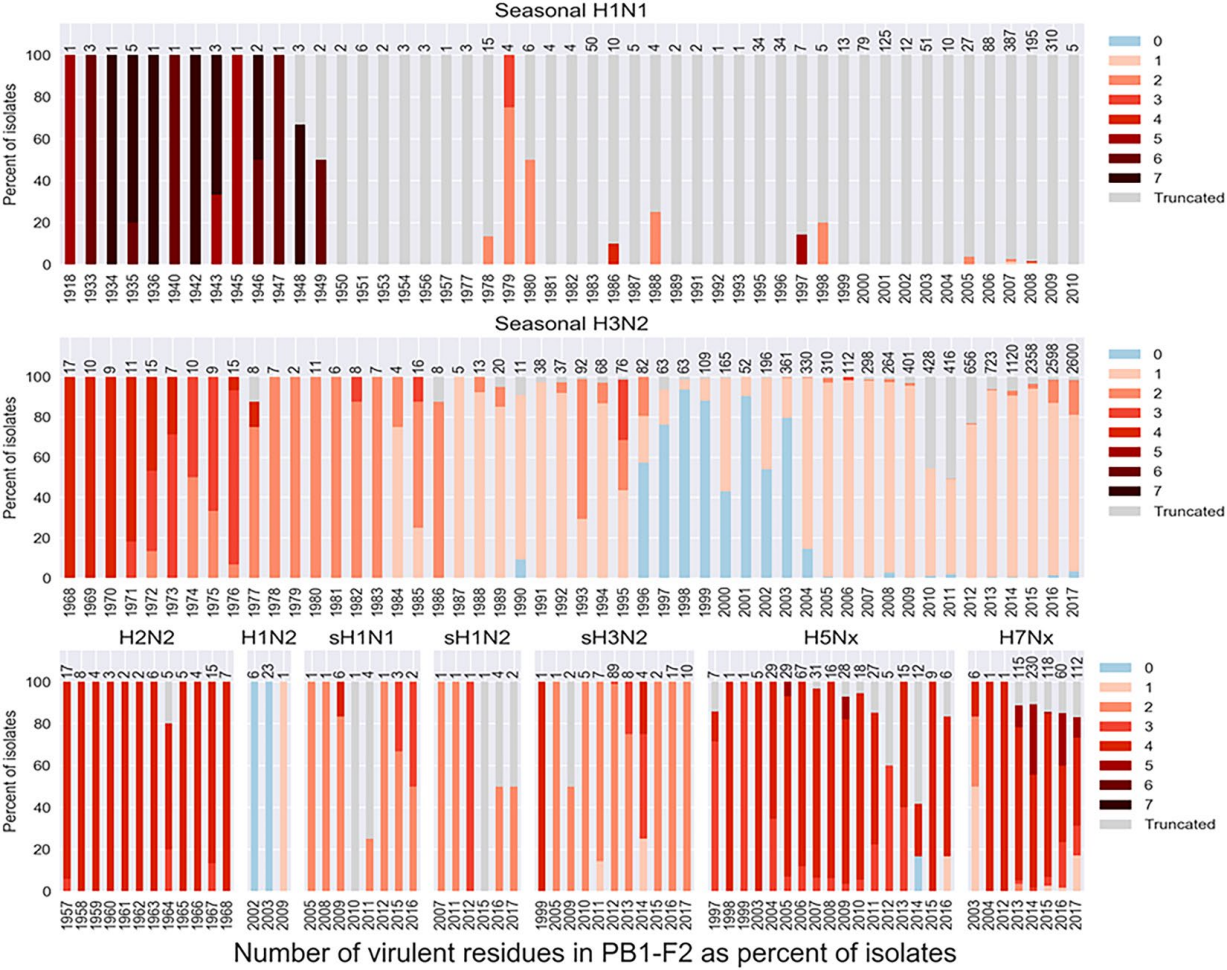
Evolution of PB1-F2 virulence-associated residues in human influenza A viruses. PB1-F2 sequences from human IAV isolates were downloaded from the IRD and GISAID databases on November 25^th^, 2017 as described in the text, and the number of virulent residues (L62, I68, L69, V70, R75, R79, and L82) were determined for each subtype per year. Isolates of the H1N1pdm09 not indicated on the graph, since none of them had virulent residues on PB1-F2 due to the presence of premature stop codon at position 12^45^. Swine origin human IAV are indicated as sH1N1, sH1N2, and sH3N2. Avian origin human IAV are indicated as H5Nx and H7Nx.

Seasonal H1N1, H2N2, and H3N2 IAVs all show a trend toward reduction in the number of virulent residues during adaptation in humans (Table 1, Fig. 1 and Supplementary Table 1), either because of PB1-F2 truncation or because of point mutations. All the 1918(H1N1) and 1968(H3N2), and sixteen of the seventeen 1957(H2N2) pandemic IAVs, had all four inflammatory residues. Seasonal H1N1 isolates with four to seven virulent residues circulated in humans until the late 1940s. After this point, H1N1 viruses tended to either have truncated PB1-F2 that were too short to include virulent residues, while those viruses that did retain a full-length PB1-F2 predominantly had only two virulent (inflammatory) residues.

H3N2 viruses arose through reassortment between avian and human IAV, and the PB1 genome segment (and therefore PB1-F2) originated from the avian lineage^46^. As with seasonal H1N1 IAVs, early seasonal H3N2 isolates (from 1968 to 1972) frequently had a full inflammatory motif of four residues. This number tended to decrease over the next 30 years, such that H3N2 isolates in the late 1970s through the early 1990s typically had two inflammatory residues. Since the late 1990s, most viruses have had only one inflammatory PB1-F2 residue. Cytotoxic residues, while rare in the first 25 years after H3N2 introduction, began to increase in frequency after the mid-1990s, especially V70, which has become very common among H3N2 isolates since 2003. Although those IAV that routinely circulated in humans (i.e. seasonal H1N1, H2N2, and H3N2) showed a trend toward loss of virulent PB1-F2 residues, IAV with zoonotic potential maintain a reservoir of virulent PB1-F2. In general, human isolates of zoonotic swine or avian IAV had a higher number of virulent residues than did seasonal IAVs. For example, over 80.0% of PB1-F2 sequences from H1N1, H1N2, and H3N2 human isolates of swine origin contained two or more virulent residues (Table 1, Fig. 1 and Supplementary Table 1).

Humans have been infected with avian IAVs of the H5Nx and H7Nx subtypes. Only 949 PB1 sequences encompassing the complete PB1-F2 ORF were available for analysis as of November 25^th^, 2017. More than 77% of such isolates with a PB1-F2 protein of 62 residues or more contained four to five virulent residues.

Combinations of inflammatory and cytotoxic residues were notably present in PB1-F2 from human isolates of swine and avian origin. Typically, these swine and avian origin viruses contained the cytotoxic residue I68 on their PB1-F2, in addition to inflammatory residues.

### Virulence of PR8(H1N1) PB1-F2-derived peptides in mice

**Figure 2:**
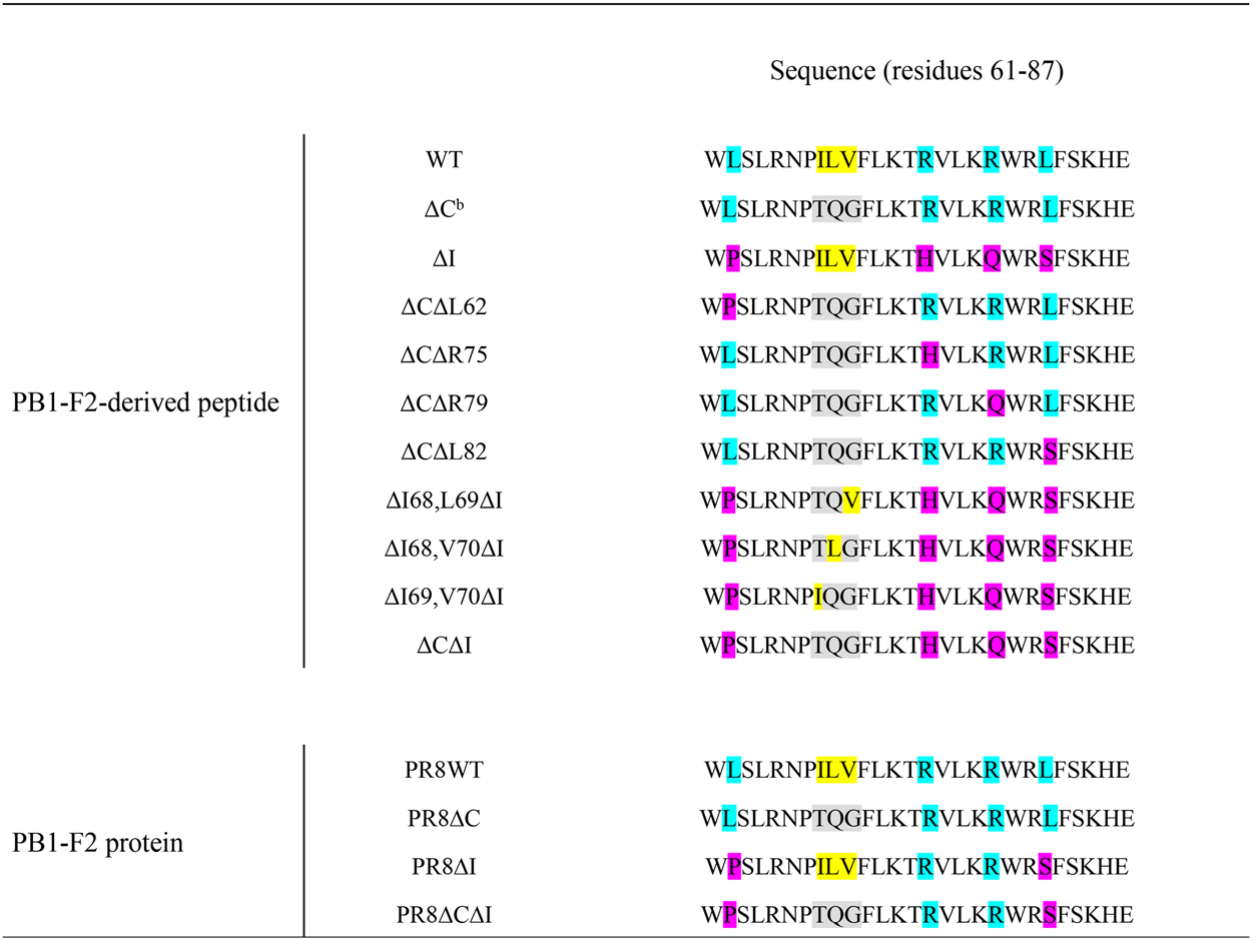
Sequences of H1N1 PB1-F2-derived peptides and corresponding proteins in reverse-genetics viruses used in these studies. Sequences are shown for 27mer C-terminal region of PB1-F2s. Yellow shading highlights the residues shown to enhance cytotoxicity in case of A/Puerto Rico/8/1934(H1N1) (PR8)^31^, and light gray shading indicates the changes to these residues. Turquoise shading indicates residues shown to enhance PB1-F2-related inflammation in the case of pandemic A/Hong Kong/1/1968(H3N2)^16^, and pink shading indicates the changes to these residues on the PR8 background.

Since PB1-F2 of contemporary human IAVs of all subtypes may possess combinations of inflammatory and cytotoxic residues, we next evaluated the effect of inflammatory residues alone or in combination with cytotoxic residues in H1N1 virulence. Chemically synthesized 27-mer C-terminal PB1-F2 peptides were shown to predict the virulence of PB1-F2 proteins with the same sequence^16,31^. We intranasally (i.n.) treated mice once with various doses of PR8 PB1-F2 wild-type (WT), lacking cytotoxic (ΔC), inflammatory (ΔI), or both motifs (ΔCΔI) peptides (peptides’ sequences are shown in Fig. 2).

**Figure 3:**
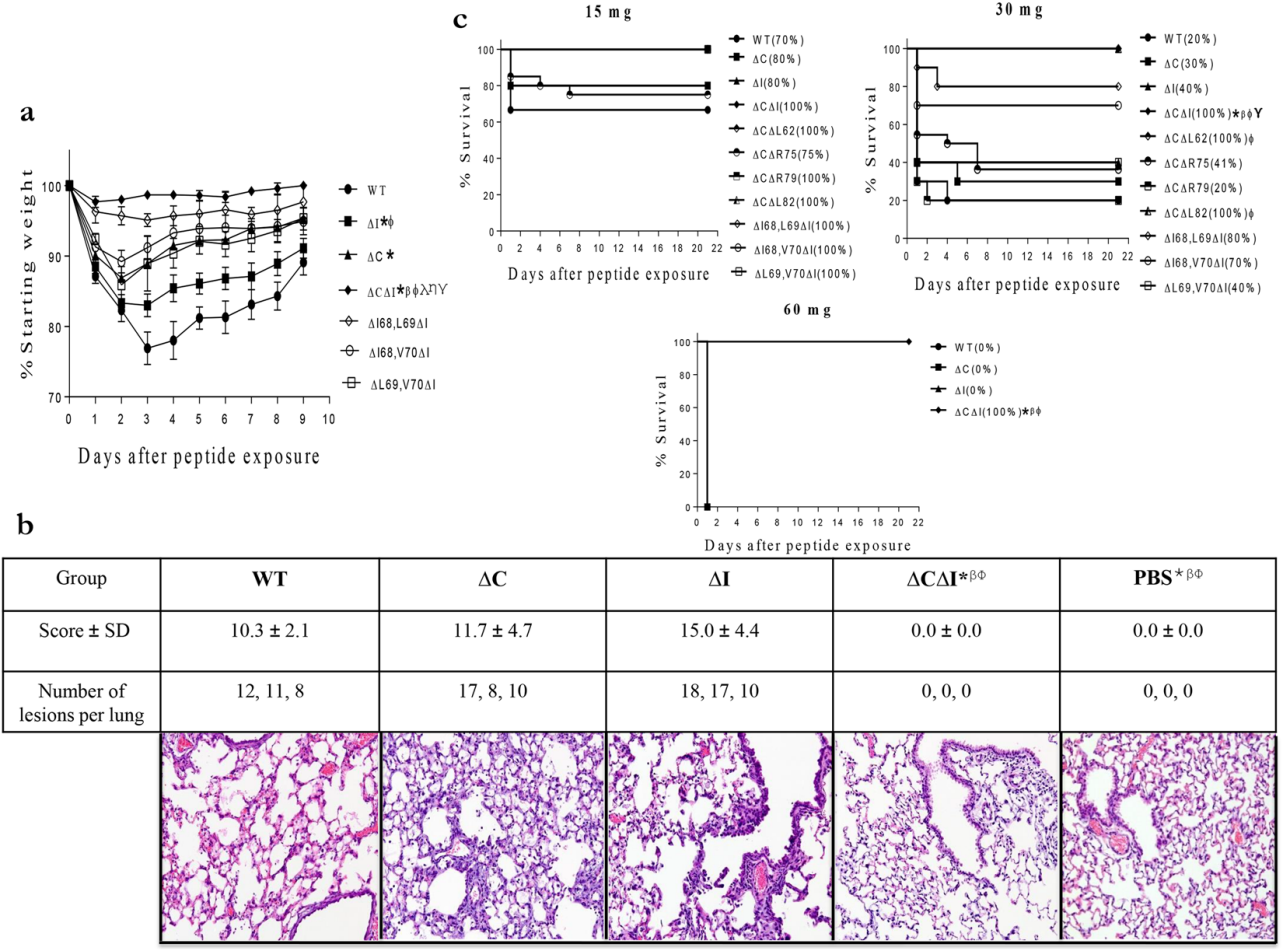
Virulence of H1N1 PB1-F2-derived peptides in mice. Weight loss (**a**) and lung histopathology (**b**) of BALB/c mice intranasally exposed to 15 mg of PR8 peptides. The mean percentage of starting weight ± SD of mice (n = 10 each group) is shown (**a**). The individual and total number of lesions per lung and scores (mean ± SD) for all pulmonary lesions for 3 mouse lungs per group were determined at day 3 after peptide administration. Stained sections of lungs are shown at 20 × magnification (**b**) Survival of mice (n = 10 each group except for the ∆C∆R75 peptide, for which 20 and 22 mice were used for dosages of 15 mg and 30 mg, respectively) exposed to various peptides dosages after 21 days of monitoring (**c**) A linear mixed model with repeated measures (**a**) and ANOVA followed by a t-test (**b**) and log-rank pairwise survival tests (**c**) (both with the Bonferroni correction for multiple comparisons) were used for comparisons. Significant differences (p ≤ 0.05) are shown as compared with the WT (*), the ΔI (β), the ∆C (Φ), ∆I68,L69ΔI (λ), ∆I68,V70ΔI (η), and ∆L69,V70ΔI (γ). (**a**) The ∆C data have been previously published (ref.^31^). ∆C– and ∆I – peptides without either cytotoxic or inflammatory motif, respectively; ΔCΔL62, ΔCΔR75, ΔCΔR79, and ΔCΔL82 – peptides without cytotoxic motif and one of the inflammatory residue; ∆I68,L69ΔI, ∆I68,V70ΔI, and ∆L69,V70ΔI – peptides without inflammatory motif and any of two cytotoxic residues; ∆CΔI – peptide without both cytotoxic and inflammatory motifs (peptide sequences are shown in Fig. 2).

Treatment with 15 mg/kg of peptides lacking one of the two virulent motifs (ΔC or ΔI) led to less weight loss than did the WT peptide (p < 0.001; Fig. 3a). However, peptide ∆I induced more weight loss than ∆C (p < 0.001), suggesting that the cytotoxic motif influences the PB1-F2 virulence to a greater extent than the inflammatory one. Peptide ΔCΔI induced the least weight loss (p < 0.001; Fig. 3a), and was the only peptide whose lung histopathology (Fig. 3b) and mortality rates at doses 30 and 60 mg/kg (Fig. 3c) significantly differed from that of the WT (p < 0.02). The absence of significant differences in lung histopathology between the WT, ΔC, or ΔI at day 3 after peptide administration, but reduced weight loss of ΔC or ΔI compared to the WT through the course of study, suggest that even if the lung damage was comparable between three peptides at this early time-point, the healing process was much faster in case of peptide without either the inflammatory or cytotoxic motif.

**Figure 4:**
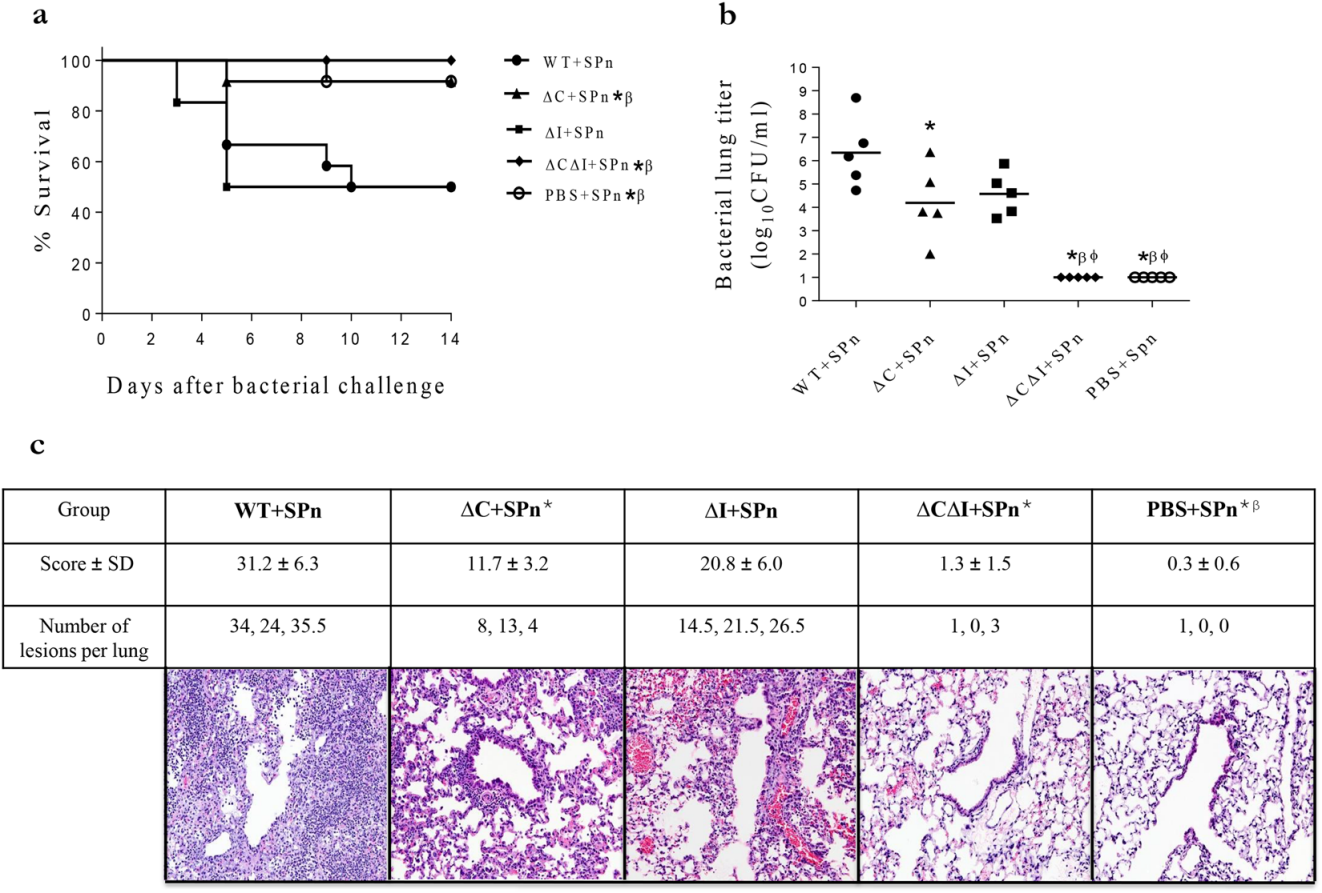
Virulence of H1N1 PB1-F2-derived peptides in a bacterial pneumonia model in mice. Mice were exposed to 10 mg of the indicated peptide and challenged 1 day later with 2,000 CFU of *S. pneumoniae* (SPn) per mouse, or with PBS. Survival of mice (n = 12 each group) (**a**) Bacterial titers in the mouse lungs (n = 5 each group) at day 1 after bacterial challenge (**b**) The individual and total number of lesions per lung and scores (mean ± SD) for all pulmonary lesions for 3 mouse lungs per group were determined at day 3 after peptide administration. Stained sections of lungs are shown at 20X magnification (**c**) A log-rank pairwise survival test (**a**) and ANOVA followed by a t-test (**b** and **c**) (both with the Bonferroni correction for multiple comparisons) were used for comparisons. Significant differences (p ≤ 0.05) are shown as compared with the WT (*), the ΔI (β), and the ∆C (Φ). (A) The ∆C data have been previously published (ref.^31^).

In a model to test susceptibility to secondary bacterial pneumonia, we treated mice with a non-lethal dose of 10 mg/kg of each PR8 PB1-F2-derived peptide, or with PBS as a control, and one day later challenged them with 2,000 colony-forming units (CFU) of *S. pneumoniae* (SPn). With this dose of SPn, more than 90% of control mice (treated with PBS instead of peptide) survived (Fig. 4a). Similarly, more than 90% and 100% of mice pre-treated with the ∆C and ∆CΔI peptides survived, respectively. The combination of the SPn with ∆I or WT peptide resulted in 50% mortality. Reflecting the general mortality trends, one day post-infection, the highest levels of geometric mean bacterial lung titers (6.4 log_10_CFU/ml) were seen in animals receiving the WT peptide, the lowest (1.0 log_10_CFU/ml) with the ∆CΔI, and the peptides containing only one of the virulent sequences ∆C and ΔI had intermediate titers (4.2 log_10_CFU/ml and 4.58 log_10_CFU/ml, respectively) (Fig. 4b).

Histopathological changes in the mouse lungs in the model of bacterial pneumonia (Fig. 4c) paralleled the mortality patterns as well, with the most severe changes seen in WT + SPn, followed by ∆I + SPn and ∆C + SPn (overall severity of 31.2 ± 6.3, 20.8 ± 6.0, and 11.7 ± 3.2 by semi-quantitative scoring, respectively). Minimal histopathological changes were observed in mice receiving ∆CΔI + SPn (overall severity of 1.3 ± 1.5; not different from PBS + SPn). Interestingly, despite similar bacterial titers in the mice receiving ∆I and ∆C, higher mortality and histopathology score were observed in ∆I group, suggesting that PB1-F2 cytotoxic motif may increase virulence through mechanisms unrelated to bacterial titers.

To determine the contribution of specific residues within the inflammatory and cytotoxic motifs to observed effects, we compared the virulence of ∆C with that of ∆C variants lacking single inflammatory residues (∆C∆L62, ∆C∆R75, ∆C∆79, or ∆C∆L82), and the virulence of ∆C∆I with that of ∆I variants also lacking two cytotoxic residues (∆I68, L69∆I, ∆I68, V70∆I, or ∆L69, V70∆I) (see peptides’ sequences in Fig. 2). As determined by mortality at 30 mg/kg of peptide, only L62 and L82 inflammatory residues enhanced the virulence of H1N1 PB1-F2-derived peptide (p < 0.001; Fig. 3c**)**. This contrasted to the four residues identified for the H3N2 subtype^16^. All three cytotoxic residues (I68, L69, and V70) contributed to the weight loss of H1N1 PB1-F2-derived peptide in mice at dosage 15 mg/kg (p < 0.001; Fig. 3a), and the I68 residue elicited the most noticeable mortality of 60% at dosage 30 mg/kg (p < 0.05; Fig. 3c).

This study confirms that specific amino acid sequences, rather than a generic peptide or administration effect, contribute to the virulence of PR8(H1N1) PB1-F2-derived peptides. The dosages and sequence combinations used allowed us to determine that two inflammatory (L62 and L82) and all three cytotoxic (I68, L69, and V70) residues contribute to virulence of this peptide, and that the presence of both types of resides enhance virulence to a higher extent than either motif alone.

### Virulence of PR8(H1N1) PB1-F2 mutant viruses in mice

We next evaluated these effects with PR8 virus variants constructed using reverse genetics (RG). BALB/c mice were infected i.n. with a sub-lethal dose of 15 PFU per mouse of PR8WT (containing all three cytotoxic and two L62 and L82 inflammatory residues within the C-terminus of PB1-F2 protein); PR8∆C (lacking all three cytotoxic residues); PR8∆I (lacking two inflammatory residues); and PR8∆C∆I (lacking all virulent residues) (the C-terminus protein sequences are shown in Fig. 2) and viral replication in the mouse lungs were determined.

**Figure 5:**
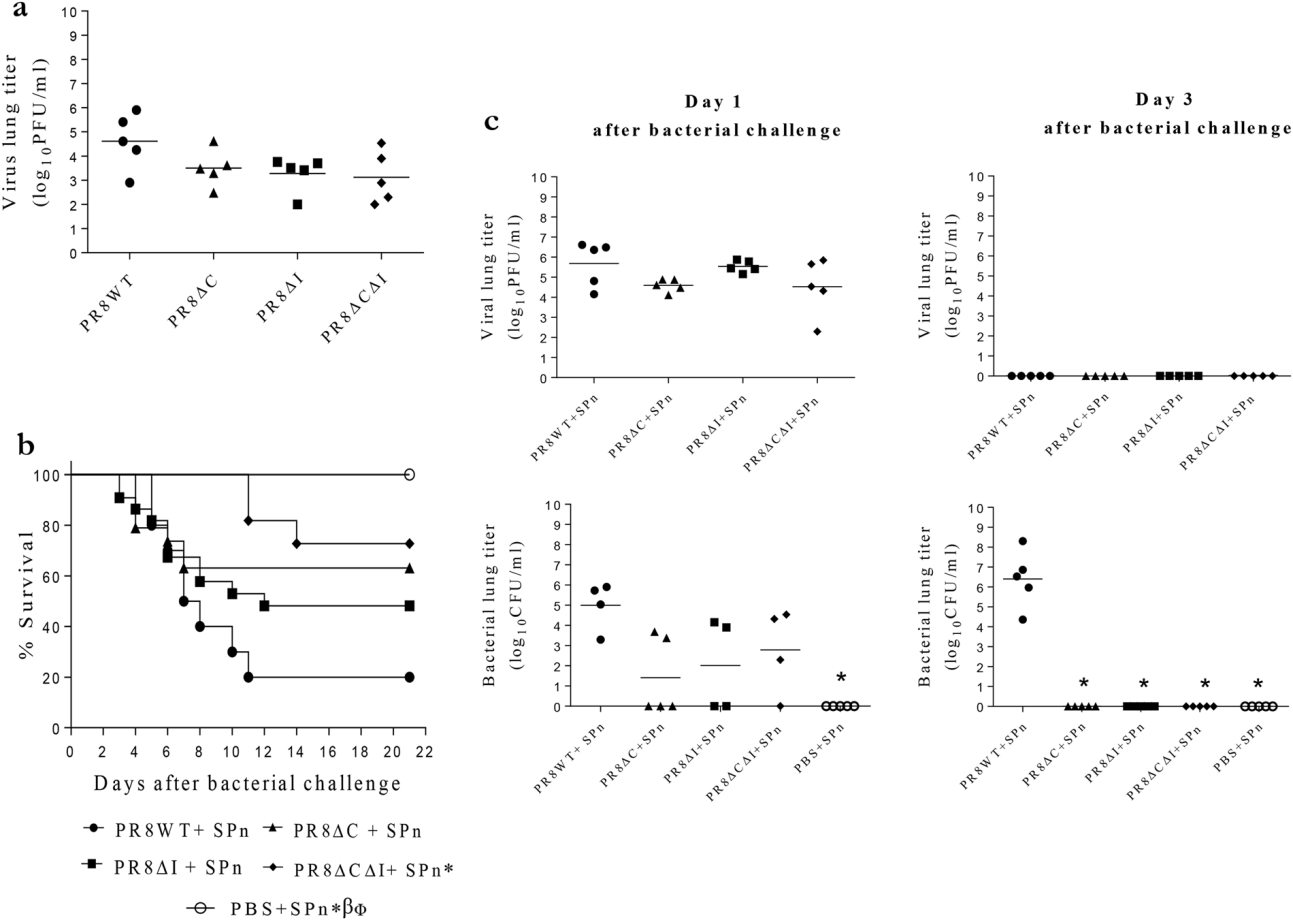
Virulence of H1N1 PB1-F2 mutant viruses in mice. BALB/c mice were intranasally infected with PR8 viruses at a sublethal dose of 15 PFU per mouse (**a**–**d**), and challenged 7 days later with a sublethal dose of 100 CFU of SPn per mouse or PBS (**b**–**d**). Virus titers in the mouse lungs (n = 5 each group) at 8 days p.i. (**a**) Survival of mice (n = 10 each group except for the PR8∆C and PR8∆I viruses, for which 19 and 22 mice were used, respectively) after bacterial challenge (**b**). Mouse lungs (n = 4–5 per group; number for each group is indicated in the figure) were collected 1 and 3 days after bacterial challenge and titrated for viral (**c**) and bacterial (**d**) loads. ANOVA followed by t-test (**a**,**c**, and **d**) and a log-rank pairwise survival test (**b**) (both with the Bonferroni correction for multiple comparisons) were used to determine the significance of differences. Significant differences (p ≤ 0.05) compared with the WT (*), the ΔI (β), and the ∆C (Φ). (C and D “Day 3 after bacterial challenge”) The ∆C data have been previously published (ref.^31^). PR8WT – virus without mutations in PB1-F2, PR8∆C – virus without cytotoxic motif (due to mutating I68T, L69Q, and V70G), PR8∆I – virus without inflammatory motif (due to mutating L62P and L82S), and PR8∆C∆I – virus without both cytotoxic and inflammatory motifs. C-terminal sequences of PR8(H1N1) PB1-F2 mutant proteins are shown in Fig. 2.

We observed no differences in the geometric mean titers between viruses up to 7 days of infection (data not shown). Notably, after 8 days p.i., titers in mice infected with PR8 mutants ranged from 3.1 log_10_PFU/ml to 3.5 log_10_PFU/ml compared to 4.6 log_10_PFU/ml of PR8WT-infected mice (Fig. 5a). This indicated the possibility that the presence of either motif may reduce the ability of the immune system to clear the virus from the lungs. There were no viruses in any group determined by 9 days p.i.

To determine the ability of PR8 PB1-F2 variants to enhance secondary bacterial pneumonia, we infected mice with a non-lethal dose of 15 PFU of virus per mouse, or treated with PBS as a control, and 7 days later challenged them with a non-lethal dose of 100 CFU of SPn per mouse or with PBS (Fig. 5b–d). There were no deaths observed in the mice given PBS instead of virus (Fig. 5b). The lowest survival (20%) was observed in the PR8WT + SPn-infected mice. Removal of either the inflammatory or cytotoxic, or both of the virulent motifs increased survival of mice to 48%, 63%, and 70%, respectively (p < 0.01 for the PR8∆C∆I group compared to WT).

One day after infection with SPn (“post-bacterial infection”: p.b.i), geometric mean viral titers in mice infected with PR8∆C or PR8∆C∆I viruses were not significantly different from those in PR8WT (about 4.5 log_10_PFU/ml compared to 5.7 log_10_PFU/ml) (Fig. 5c). There were also no differences in viral kinetics observed between groups of mice administered with PBS (instead of bacteria) and virus alone (not shown). All viruses were cleared from the mouse lungs by 3 days p.b.i. As to bacterial lung titers at 1 day p.b.i., infection of mice with PR8 PB1-F2 mutant viruses resulted in geometric mean bacterial titers ranging from 1.4 log_10_CFU/ml to 2.8 log_10_CFU/ml compared to 5.0 log_10_CFU/ml in mice infected with PR8WT (Fig. 5c). All mice infected with mutant viruses cleared bacteria from the lungs by 3 day p.b.i. (5 C). However, mice in the PR8WT-infected group still had geometric mean bacterial titers 6.4 log_10_CFU/ml at this time-point (p < 0.001; Fig. 5c). There were no bacteria observed in the PBS + SPn group at these times.

The PB1-F2 mutant viruses therefore recapitulated the effects seen with synthetic PB1-F2 peptides, indicating the ability of two inflammatory residues L62 and L82 to increase virulence in the H1N1 as well as the H3N2 subtype. In addition, combining inflammatory and cytotoxic sequences in PB1-F2 increased virulence still further.

## Discussion

Mortality in humans due to IAV infection ranges widely, from 0.001%-0.007% for H1N1pdm2009 to 39%-53% for H7N9 and H5N1 avian IAV^47,48^. Human seasonal influenza generally has a relatively low mortality rate, but can be fatal in healthy adults for several reasons, including secondary bacterial pneumonia^49^. The reasons for these variations are not well understood, but might include the IAV accessory protein PB1-F2, a protein known for its sequence-dependent virulence^16,31^.

Certain residues in PB1-F2 of some IAVs have inflammatory or cytotoxic activities^10,19^. In the context of H3N2 PB1-F2, residues L62, R75, R79, and L82 in PB1-F2 increase the inflammatory effects of synthetic PB1-F2-derived peptides and RG HK68 viruses, resulting in promotion of secondary bacterial pneumonia in mice^16^. The effect of PB1-F2 on IAV virulence varies widely depending on virus strain. We determined the ability of two inflammatory residues (L62 and L82) to induce virulent effects in the context of the mouse-adapted laboratory strain PR8(H1N1) PB1-F2 (Figs 3–5). Our data suggest that the effects of residues within the inflammatory motif might be applicable for any IAV. This proposition is supported by recent studies showing enhancement of inflammation, as a result of the NLRP3 inflammasome activation, by the presence of this motif in H7N9 PB1-F2^17^.

All three I68, L69, and V70 residues within the previously described cytotoxic motif ^31^ increased the PR8(H1N1) PB1-F2 virulence (Fig. 3a). Altered peptide experiments (Figs 3a and 4a) suggest that the cytotoxic motif is more virulent than the inflammatory one. The combined presence of both the inflammatory (L62 and L82) and cytotoxic (I68, L69, and V70) residues in PR8(H1N1) PB1-F2 resulted in the most pathogenic phenotype of either peptide or protein, suggesting that the effects of each motif are additive or synergistic.

It is important to note that peptide without virulent residues, ΔCΔI, did not induce any toxicity or virulence at any tested dose in mice (Fig. 3a–c). This is consistent with our previous studies showing that cells in tissue culture showed no toxic or virulent effects when exposed either to PB1-F2 peptide variants derived from H1N1 PR8 or H3N2 A/Wuhan/359/1995 that lacked virulence residues^31^. Together, our data demonstrate that peptide per se is not toxic in tissue culture or in mice at the doses tested, and it is the presence of specific residues in the peptides that causes adverse effects.

Each of the major twentieth-century influenza pandemics of 1918(H1N1), 1957(H2N2), and 1968(H3N2) included a PB1 segment that encodes full-length PB1-F2 with all four inflammatory residues; the pandemic 1918 PB1-F2 also had a cytotoxic residue, V70 (Fig. 1 and Supplementary Table 1). In each case, the PB1 segment was of avian origin, and was presumably not optimally adapted for replication in mammals. In the years following their introduction into humans, the PB1-F2 of H1N1 and H3N2 strains showed a reduction of the number of virulence-associated residues, either through the mechanism of PB1-F2 truncation (as in H1N1) or through substitutions by non-virulent residues (as with H3N2). We previously proposed that these changes in PB1-F2 result in reduced protein virulence in humans^16^. Recently, this proposition was experimentally confirmed for the H3N2 inflammatory motif ^50^. The loss of virulence motifs may reflect adaptation of the viruses to transmission and replication in humans, or to avoidance of immune surveillance.

The PB1-F2 S66 polymorphism also has been linked to the enhanced virulence of pandemic 1918(H1N1) and HPAIV(H5N1)^12,13^. Similarly to the inflammatory and cytotoxic motifs, S66 began to disappear from H1N1 following its introduction into humans, and is now only occasionally observed in some human seasonal H3N2, and in human isolates of avian origin H5Nx and H7Nx IAV (Supplementary Table 1), further suggesting that loss of PB1-F2 virulence as IAV adapts to humans is a general trend.

As the proportion of PB1-F2 lacking virulent residues in human seasonal IAVs has risen, rare exceptions have occurred. For example, one 1997 H1N1 IAV (A/Taiwan/3355/97; Supplementary Table 1) possessed two inflammatory residues (L62 and L82) and a full cytotoxic motif. This virus was isolated from a patient with severe pneumonia^51^, which is uncommon in H1N1 infections, consistent with the notion that the combined sequences lead to increased virulence.

Avian and swine influenza viruses sporadically infect humans and have potential for transient or sustained human-to-human transmission, as well as for reassortment with seasonal human viruses^52–54^. Human zoonotic viruses typically contained more virulent PB1-F2 than did the contemporary seasonal strains (Fig. 1 and Supplementary Fig. 1), with up to four virulent residues being present in human swine IAV and as many as seven in human avian IAV isolates. These viruses therefore represent a reservoir of PB1-F2 proteins that could contribute increased virulence to human-transmitted IAV.

The association of these residues with virulence of pandemic 1918, 1957, and 1968 IAV and HPAIV, and the consistent and marked effects of these residues on the virulence of several strains of IAV in mice, suggests that assessment of virulence-associated residues in circulating IAV might be useful for predicting the severity of influenza seasons. The potential for zoonotic reservoirs to donate virulence-associated PB1-F2 to the human pool of viruses reinforces the importance of both human and animal surveillance.

## Methods

### Database analysis of virulent PB1-F2 residues

PB1 segment nucleotide sequences of H1N1, H1N2, H2N2, H3N2, H5 and H7 human isolates from the Global Initiative on Sharing All Influenza Data (http://platform.gisaid.org; acknowledged in Supplementary Table 2) and Influenza Resource Database (IRD; https://www.fludb.org/brc/home.spg?decorator=influenza) were assessed on November 25^th^, 2017. Within each subtype, both databases sequences were merged, eliminating virus strain name duplicates. The year of collection and virus origin (swine or avian in case of cross-species transmission) were noted. PB1 sequences were aligned using MUSCLE in IRD, with manual deletion of gaps. Inconclusive sequences (those not covering complete PB1-F2 ORF or from an ambiguous source) were excluded from analyses. Sequences without an initiating ATG codon at the appropriate position were excluded from counts. The PB1-F2 protein sequences were sorted into two groups (of 62 amino acids or longer and 61 amino acid or shorter) using BioEdit (v7.1.3.0). Sequences of 62 amino acid or longer were saved as MEGA files, displayed and exported to MS EXCEL. The inflammatory (L62, R75, R79, and L82) or/and cytotoxic (I68, L69, and V70) PB1-F2 residues were highlighted, and the sequences were sorted by the presence of residue(s) and year of isolation.

### PB1-F2 peptides

Using the predicted amino acid sequence of the A/Puerto Rico/8/1934(H1N1) (“PR8”) PB1-F2, the 27-mer C-terminus (residues 61–87) PB1-F2-derived peptides with no, single, or multiple substitutions at positions 62, 68, 69, 70, 75, 79, and 82 were synthesized (GenScript Corp., Piscataway, NJ) (Fig. 2). Mutations (Ile 68 to Thr [I68T], Leu 69 to Gln [L69Q], and Val 70 to Gly [V70G]) to the wild-type peptide (WT) were made to eliminate the cytotoxic motif as previously described^31^. This peptide was designated ∆C. Similarly, mutations (Leu 62 to Pro [L62P], Arg 75 to His [R75H], Arg 79 to Gln [R79Q], and Leu 82 to Ser [L82S]) to the WT were made to eliminate the inflammatory motif found naturally in 1968(H3N2)^16^. This peptide was designated ΔI, and the peptide lacking both virulent motifs was designated ΔCΔI. The intermediate variants (∆C∆L62, ∆C∆R75, ∆C∆79 or ∆C∆L82) missing a full cytotoxic motif and a single inflammatory residue had mutations I68T, L69Q, V70G, and L62P, R75H, R79Q, or L82S, respectively. The intermediate versions (∆I68,L69∆I; ∆I68,V70∆I; ∆L69,V70∆I) lacking a full inflammatory motif and any of two cytotoxic residues had mutations I68T, L69Q; or I68T,V70G; or L69Q,V70G; and L62P, R75H, R79Q, and L82S. Lyophilized peptides were utilized as described^16,31^.

### Viruses and bacteria

PR8 and its mutant variants were generated by reverse genetics (RG) as described previously^31^. The PB1 gene segment was modified to generate PR8 variants with I68T, L69Q, and V70G mutations in the PB1-F2 ORF to remove the cytotoxic motif (PR8ΔC), or with L62P and L82S mutations to remove the inflammatory motif (PR8ΔI), or with all seven mutations to eliminate both the cytotoxic and inflammatory motifs (Fig. 2). The mutations in PB1-F2 were designed so as not to cause non-synonymous changes in the PB1. The full genomes of Madin-Darby canine kidney (MDCK) cells grown viruses were sequenced to confirm the absence of undesired mutations.

*Streptococcus pneumoniae* strain A66.1 type 3 (SPn) was grown and titrated as previously described^16,31^.

### Studies in mice

Experiments using 8-week-old female BALB/c mice (Jackson Laboratories, Bar Harbor, ME) were performed in a Biosafety Level 2 facility in the Animal Resources Center at St. Jude Children’s Research Hospital (St. Jude; Memphis, TN), approved by the institutional animal care and use committee at St. Jude and performed in accordance with the relevant guidelines and regulations. Animals were given general anesthesia that consisted of 2.5% inhaled isoflurane (Baxter Healthcare Corporation, Deerfield, IL) prior to all interventions.

The virulence of PB1-F2-derived peptides was determined in mice (at least 10 per group) that received 15, 30, or 60 mg peptide once i.n. in a volume of 100 μl. In the bacterial pneumonia study, mice were exposed to 10 mg per mouse of peptides or to PBS (control group) and 1 day later were challenged with 2,000 colony-forming units (CFU) of SPn per mouse. Weight changes (calculated for each mouse as a percentage of its weight on day 0 before peptide administration) and survival of mice were monitored for 21 days after peptide administration. Survival of mice in a bacterial pneumonia model was monitored for 14 days after bacterial challenge. For bacterial titers, lungs of 5 mice per group were collected 1 day after bacterial challenge.

The growth of RG PR8 PB1-F2 virus variants was compared in mice infected i.n. with 15 plaque-forming units (PFU) per mouse in 100 μl PBS. For quantification of virus titers, lungs from 3 to 5 mice were harvested at days 1, 3, 5, 7, 8, or 9 post-infection (p.i.). In the secondary bacterial pneumonia study, mice were infected with 15 PFU of PB1-F2 virus variants or given PBS (control group) and challenged 7 days later with 100 CFU of SPn per mouse. Survival of mice was monitored for 21 days after the bacterial challenge. Viral and bacterial titers in the mouse lungs (n = 4–5 per group) were determined 1 and 3 days after bacterial challenge. The lung pathology was evaluated in 3 mice per group 3 days post-bacterial infection (p.b.i.) in case of both peptide and virus studies.

### Viral and bacterial lung titers

Mouse lungs were homogenized, suspended in 1 ml PBS, and centrifuged at 2,000 × *g* for 10 minutes to clear cellular debris for virus titration, or were used without centrifugation for bacterial cultures. Ten-fold dilutions were used to determine the virus titers by plaque assays in MDCK cells^55^ or pneumococcal colony counts on tryptic soy agar plates.

### Histopathology of mouse lungs

Lungs were processed for hematoxylin and eosin staining, and examined microscopically by an experienced veterinary pathologist (P.V.) in a single-blinded manner as previously described^31^. The pulmonary lesions that were evaluated and scored included bronchiolitis (necrosis, inflammation, and plugging), bronchiolar epithelial proliferation, bronchiolar denudation, peribronchiolar edema and inflammation, vascular margination and cuffing, alveolitis (necrosis and inflammation), alveolar hemorrhage/edema/fibrin, alveolar epithelial hyperplasia/hypertrophy, interstitial inflammation, interstitial fibrosis, alveolar bronchiolization, lymphoid nodule formation, pulmonary consolidation, and overall extent. The severity of pulmonary lesions were evaluated independently and graded as follows: 1 = minimal, focal to multifocal, inconspicuous; 2 = mild, multifocal, conspicuous; 3 = moderate, multifocal, prominent; 4 = marked, multifocal coalescing, lobar; 5 = severe, extensive, diffuse, with consolidation. The overall severity score for each lung was obtained by adding all of the individual lesion scores, and average group scores were calculated from these totals.

### Statistical analysis

Analysis of variance (ANOVA) followed by a t-test with the Bonferroni correction for multiple comparisons was used to compare the viral and bacterial titers in mouse lungs, and histopathologic scores. A linear mixed model with repeated measures was used to compare weight loss. Survival between groups of mice was compared by the log-rank pairwise test with the Bonferroni correction for multiple comparisons to analyze survival data over the period of 14 or 21 days. The mean number of days until death was estimated as the number of days that the mice survived after peptide administration or viral or bacterial infection. A p value ≤0.05 was considered significant for all comparisons. Statistical analyses were performed using GraphPad Prism Software (v5.0, GraphPad Software Inc., San Diego, CA), the “lifelines” package in Python (v0.14), and the R programming language.

## Electronic supplementary material

Supplementary Table 1

Supplementary Dataset 1
