# Supplementary material for: Virulent PB1-F2 residues: effects on fitness of H1N1 influenza A virus in mice and changes during evolution of human influenza A viruses
Source: Sci Rep. 2018 May 10;8:7474. doi: 10.1038/s41598-018-25707-y (PMC5945659; doi:10.1038/s41598-018-25707-y)
Supplement: Supplementary file 1 — Supplementary Table 1 [file 41598_2018_25707_MOESM1_ESM.pdf]

Virulent PB1-F2 residues: effects on fitness of H1N1 influenza A virus in mice and changes during evolution of human influenza A viruses

Running title: influenza A PB1-F2 virulence

Irina V. Alymova,[1] Jonathan A. McCullers, [2] Ram P. Kamal,[1,5] Peter Vogel,[4] Amanda M. Green,[3a] Shane Gansebom,[3b] & Ian A. York[1]

[1]Influenza Division, National Center for Immunization & Respiratory Diseases, Centers for Disease Control & Prevention, Atlanta, GA USA; [2]Department of Pediatrics, University of Tennessee Health Sciences Center, Memphis, TN USA; Departments of [3]Infectious Diseases and [4]Pathology, St. Jude Children's Research Hospital, Memphis, TN USA; [5]Battelle Memorial Institute, Atlanta, GA USA

Correspondence and requests for materials should be addressed to IVA (email: xeq3@cdc.gov)

Present address: [a]Department of Graduate Medical Education, University of Tennessee Health Sciences Center, Memphis, TN USA; [b]Influenza Division, National Center for Immunization & Respiratory Diseases, Centers for Disease Control & Prevention, Atlanta, GA USA, and CNI Advantage, LLC, Norman, OK USA

Human seasonal H1N1 isolates

Isolation year	Presence of virulent residues at positions								No. of isolates	No. of isolates per year				
	62	66	68	69	70	75	79	82		≥62 amino acids			≤61 amino acid	Total[β]
										with virulent residues[α]	with virulent residue S66	without any virulent residue		
2011-17									0	0	0	0	11,676	11,676
2010										0	0	0	5	5
2009										0	0	0	310	310
2008					V				1	3	0	0	192	195
	L							L	1					
	L		I				R	L	1					
2007					V				5	10	0	0	377	387
	L					R			1					
	L							L	4					
2006										0	0	0	88	88
2005	L							L	1	1	0	0	26	27
2004										0	0	0	10	10
2003										0	0	0	51	51
2002										0	0	0	12	12
2001										0	0	0	125	125
2000										0	0	0	79	79
1999										0	0	0	13	13
1998	L							L	1	1	0	0	4	5
1997	L		I	L	V			L	1	1	0	0	6	7
1996										0	0	0	34	34
1995										0	0	0	34	34
1994										No isolate				
1993										0	0	0	1	1
1992										0	0	0	1	1

**1991**									0	0	0	2	2
**1990**									No isolate				
**1989**									0	0	0	2	2
**1988**	L				R			1	1	0	0	3	4
**1987**									0	0	0	5	5
**1986**	L				R	R	L	1	1	0	0	9	10
**1985-84**									No isolate				
**1983**									0	0	0	50	50
**1982**									0	0	0	4	4
**1981**									0	0	0	4	4
**1980**	L				R			3	3	0	0	3	6
**1979**	L				R			3	4	0	0	0	4
	L				R		L	1					
**1978**	L				R			2	2	0	0	13	15
**1977**									0	0	0	3	3
**1976-58**									No isolate				
**1957**									0	0	0	1	1
**1956**									0	0	0	3	3
**1955**									No isolate				
**1954**									0	0	0	3	3
**1953**									0	0	0	2	2
**1952**									No isolate				
**1951**									0	0	0	6	6
**1950**									0	0	0	2	2
**1949**	L	I	L	V	R		L	1	1	0	0	1	2
**1948**	L	I	L	V	R	R	L	2	2	0	0	1	3
**1947**	L	I	L	V	R		L	1	1	0	0	0	1
**1946**	L	I	L	V	R		L	1	2	0	0	0	2
	L	I	L	V	R	R	L	1					
**1945**	L		L	V		R	L	1	1	0	0	0	1
**1944**									No isolate				
	L	I	L	V	R	R	L	2					

**1943**	L			L				L	1	3	0	0	0	3
**1942**	L		I	L	V	R	R	L	1	1	0	0	0	1
**1941**	No isolate													
**1940**	L		I	L	V	R		L	1	1	0	0	0	1
**1939-37**	No isolate													
**1936**	L		I	L	V	R	R	L	1	1	0	0	0	1
**1935**	L		I	L	V	R	R	L	4	5	0	0	0	5
			I	L	V	R	R	L	1					
**1934**	L		I	L	V	R	R	L	1	1	0	0	0	1
**1933**			I	L	V	R	R	L	3	3	0	0	0	3
**pdm1918**	L	S			V	R	R	L	1	1	1	0	0	1
**Total**									**50**	**50**	**1**	**0**	**13,191**	**13,211**

Human H1N1 isolates of swine origin

**Isolation year**	**Presence of virulent residues at positions**								**No. of isolates**	**No. of isolates per year**				
	62	66	68	69	70	75	79	82		**≥62 amino acids**			**≤61 amino acid**	**Total[β]**
										**with virulent residues[α]**	**with virulent residue S66**	**without any virulent residue**		
**2016**	L		I						1[a]	2	0	0	0	2
	L		I					L	1[a]					
**2015**	L								1[b]	3	0	0	0	3
	L		I			R			1[b]					
	L								1[b]					
**2014**										0	0	0	1[b]	1
**2013**	No isolate													
**2012**	L							L	1[b]	1	0	0	0	1
**2011**	L							L	1[b]	1	0	0	2[a]	3

Isolation year	62	66	68	69	70	75	79	82	No. of isolates	≥62 amino acids with virulent residues[α]	≥62 amino acids with virulent residue S66	≥62 amino acids without any virulent residue	≤61 amino acid	Total[β]
**2010**										0	0	0	1[a]	1
**2009**	L							L	3[c]	6	0	0	0	6
	L							L	2[b]					
	L		I				R	L	1[a]					
**2008**	L							L	1[b]	1	0	0	0	1
**2007-06**										No isolate				
**2005**	L							L	1[b]	1	0	0	0	1
**Total**									**15**	**15**	**0**	**0**	**4**	**19**

Human seasonal H1N2 isolates

Isolation year	Presence of virulent residues at positions 62	66	68	69	70	75	79	82	No. of isolates	No. of isolates per year: ≥62 amino acids with virulent residues[α]	≥62 amino acids with virulent residue S66	≥62 amino acids without any virulent residue	≤61 amino acid	Total[β]
**2009**					V				1	1	0	0	0	1
**2003**									0	0	0	23	0	23
**2002**									0	0	0	6	0	6
**Total**									**1**	**1**	**0**	**29**	**0**	**30**

Human H1N2 isolates of swine origin

Isolation year	Presence of virulent residues at positions 62	66	68	69	70	75	79	82	No. of isolates	No. of isolates per year: ≥62 amino acids with virulent residues[α]	≥62 amino acids with virulent residue S66	≥62 amino acids without any virulent residue	≤61 amino acid	Total[β]
**2017**	L							L	1	1	0		1	2

**2016**	L							L	2	2	0	0	2	4
**2015**									0	0	0	0	1	1
**2014-13**	No isolate													
**2012**	L		I				R		1	1	0	0	0	1
**2011**	L							L	1	1	0	0	0	1
**2010-08**	No isolate													
**2007**	L							L	1	1	0	0	0	1
**Total**									**6**	**6**	**0**	**0**	**4**	**10**

Human seasonal H2N2 isolates

Isolation year	Presence of virulent residues at positions								No. of isolates	No. of isolates per year				
	62	66	68	69	70	75	79	82		≥62 amino acids			≤61 amino acid	Total[β]
										with virulent residues[α]	with virulent residue S66	without any virulent residue		
**1968**	L					R	R	L	7	7	0	0	0	7
**1967**	L					R	R	L	12	15	4	0	0	15
	L	S				R	R	L	4					
	L					R	R		1					
	L					R		L	1					
**1966**	L					R	R	L	4	4	0	0	0	4
**1965**	L					R	R	L	5	5	0	0	0	5
**1964**	L					R	R	L	3	4	0	0	1	5
	L					R	R		1					
**1963**	L					R	R	L	6	6	0	0	0	6
**1962**	L					R	R	L	2	2	0	0	0	2
**1961**	L					R	R	L	2	2	0	0	0	2
**1960**	L					R	R	L	3	3	0	0	0	3

**1959**	L					R	R	L	4	4	0	0	0	4
**1958**	L					R	R	L	8	8	0	0	0	8
**pdm1957**	L					R	R	L	16	17	0	0	0	17
	L					R		L	1					
**Total**									**77**	**77**	**4**	**0**	**1**	**78**

Human seasonal H3N2 isolates

Isolation year	Presence of virulent residues at positions								No. of isolates	No. of isolates per year				
	62	66	68	69	70	75	79	82		≥62 amino acids			≤61 amino acid	Total[β]
										with virulent residues[α]	with virulent residue S66	without any virulent residue		
**2017**					V				2018	2474	1	83	43	2600
					V	R			435					
			I		V				7					
						R			5					
			I		V	R			3					
					V			L	3					
			I		V		R	L	1					
					V	R		L	1					
		S			V				1					
**2016**					V				2225	2527	0	33	38	2598
					V	R			287					
			I		V				4					
			I		V	R			4					
					V			L	3					
	L				V				2					
				L	V				1					

	L			V		R		1					
2015				V				2213	2266	0	4	88	2358
				V	R			37					
	L			V				6					
			I	V				5					
				V			L	4					
			I	V	R			1					
2014				V				1006	1039	4	4	77	1120
				V			L	14					
				V	R			9					
	L			V				4					
		S		V				4					
			I	V				1					
	L							1					
2013				V				670	676	3	1	44	723
		S						2					
				V		R		2					
		S		V				1					
			I	V				1					
				V	R			1					
	L			V				1					
2012				V				501	504	0	0	152	656
				V		R		1					
			I	V				1					
				V	R			1					
2011				V				195	197	0	8	211	416
	L							1					
	L			V				1					
2010				V				229	229	1	3	195	421
		S						1					
				V				380					
				V	R			3					

**2009**	L			V				2	388	2	1	12	401
		S		V				2					
			I	V				1					
**2008**				V				240	254	13	4	3	264
		S		V				10					
				V			L	3					
		S						3					
	L			V				1					
**2007**				V				291	293	0	1	4	298
				V	R			1					
				V			L	1					
**2006**				V				110	112	0	0	0	112
	L			V	R			2					
**2005**				V				298	306	1	2	2	310
				V			L	3					
			I	V				2					
				V	R			1					
	L			V				1					
		S		V				1					
**2004**				V				278	281	1	48	1	330
				V			L	1					
						R		1					
		S		V				1					
**2003**				V				69	73	11	276	1	361
		S						11					
							L	2					
				V			L	1					
						R		1					
**2002**							L	85	89	1	105	1	196
				V				4					
		S						1					
				V				3					

			I					1					
**2001**							L	1	5	0	47	0	52
	L							89					
**2000**				V				4	93	1	70	1	165
		S						1					
						R		6					
		S				R		3					
**1999**				V				1	12	3	96	1	109
			I					1					
	L							1					
						R		2					
**1998**							L	1	3	0	59	1	63
**1997**	L							11	11	0	48	4	63
	L							17					
**1996**	L						L	16	35	1	47	0	82
		S				R		1					
				V				1					
	L							33					
**1995**	L		I				L	23	75	0	0	1	76
	L						L	19					
	L							120					
**1994-92**	L						L	73	193	0	0	4	197
**1991-90**	L							46	46	0	1	2	49
	L							16					
**1989**	L				R			2	19	1	0	1	20
	L	S						1					
	L				R			12					
**1988**	L							1	13	0	0	0	13
**1987**	L							5	5	0	0	0	5
**1986**	L				R			7	7	0	0	1	8
	L				R			10					
					R			4					

**1985**	L			R	R		2	16	0	0	0	16
**1984**				R			3	4	0	0	0	4
	L			R			1					
**1983**	L			R			6	7	1	0	0	7
	L	S		R			1					
**1982**	L			R			7	8	0	0	0	8
	L			R	R		1					
**1981-78**	L			R			26	26	0	0	0	26
**1977**	L			R			6	7	0	0	1	8
	L			R	R	L	1					
**1976**	L			R	R		13	15	0	0	0	15
	L			R			1					
	L		I	R	R		1					
**1975**	L			R	R		6	9	0	0	0	9
	L			R			3					
**1974**	L			R	R		5	10	0	0	0	10
	L			R			4					
				R	R		1					
**1973**	L			R	R		5	7	0	0	0	7
	L			R	R	L	2					
**1972**	L			R	R		6	15	0	0	0	15
	L			R	R	L	5					
				R	R		2					
	L	I		R	R		2					
**1971**	L			R	R	L	9	11	0	0	0	11
	L			R		L	2					
**1970-69**	L			R	R	L	19	19	0	0	0	19
**pdm1968**	L			R	R	L	17	15	0	0	0	15
**Total**							**12,415**	**12,396**	**45**	**941**	**889**	**14,245**

Human H3N2 isolates of swine origin

Isolation year	Presence of virulent residues at positions: 62	66	68	69	70	75	79	82	No. of isolates	No. of isolates per year: ≥62 amino acids, with virulent residues[α]	≥62 amino acids, with virulent residue S66	≥62 amino acids, without any virulent residue	≤61 amino acid	Total[β]
2017	L							L	10[d]	10	0	0	0	10
2016	L						R		12[d]	17	0	0	0	17
	L							L	5[d]					
2015	L							L	2[d]	2	0	0	0	2
2014	L					R	R		1[d]	4	0	0	0	4
	L		I				R	L	1[e]					
	L					R		L	1[d]					
	L								1[d]					
2013	L							L	6[d]	8	0	0	0	8
	L		I				R		1[d]					
	L		I					L	1[d]					
2012	L							L	88[d]	89	0	0	0	89
	L						R	L	1[d]					
2011	L							L	6[d]	7	0	0	0	7
	L								1[d]					
2010-09	L							L	6[d]	6	0	0	1	7
2008-06									No isolate					
2005	L							L	1[d]	1	0	0	0	1
2004-00									No isolate					
1999	L					R	R	L	1[e]	1	0	0	0	1
**Total**									**145**	**145**	**0**	**0**	**1**	**146**

Human H5N1 and H5N6 isolates of avian origin

Isolation year	Presence of virulent residues at positions								No. of isolates	No. of isolates per year				
										≥62 amino acids			≤61 amino acid	Total[β]
	62	66	68	69	70	75	79	82		with virulent residues[α]	with virulent residue S66	without any virulent residue		
2017										No isolate				
2016	L					R	R	L	4	5	0	0	1	6
						R			1					
2015	L					R	R	L	9	9	0	0	0	9
2014	L					R	R	L	3	3	0	2	7	12
2013	L					R	R	L	9	15	0	0	0	15
	L						R	L	5					
	L					R	R		1					
2012	L	S					R	L	3	3	3	0	2	5
2011	L						R	L	17	23	3	0	4	27
	L	S					R	L	3					
	L						R	L	3					
2010	L					R	R	L	16	17	1	0	1	18
	L	S					R	L	1					
2009	L					R	R	L	22	26	0	0	2	28
	L		I			R	R	L	3					
	L					R	R		1					
2008	L					R	R	L	15	16	0	0	0	16
	L						R	L	1					
2007	L					R	R	L	28	30	0	0	1	31
	L					R	R		2					
	L					R	R	L	59					

Isolation year	62	66	68	69	70	75	79	82	No. of isolates	≥62 amino acids: with virulent residues[α]	≥62 amino acids: with virulent residue S66	≥62 amino acids: without any virulent residue	≤61 amino acid	Total[β]
2006	L						R	L	5	67	0	0	0	67
	L					R		L	2					
	L					R	R		1					
2005	L					R	R	L	25	29	0	0	0	29
	L					R		L	2					
	L		I			R	R	L	2					
2004	L					R	R	L	19	29	0	0	0	29
	L					R		L	10					
2003	L					R	R	L	5	5	0	0	0	5
2002-00										No isolate				
1999	L						R	L	1	1	0	0	0	1
1998	L					R	R	L	1	1	0	0	0	1
1997	L						R	L	3	6	2	0	1	7
	L	S					R	L	2					
	L					R	R	L	1					
**Total**									**285**	**285**	**9**	**2**	**19**	**306**

Human H7N2, H7N3, H7N7, and H7N9 isolates of avian origin

Isolation year	Presence of virulent residues at positions: 62	66	68	69	70	75	79	82	No. of isolates	No. of isolates per year: ≥62 amino acids: with virulent residues[α]	≥62 amino acids: with virulent residue S66	≥62 amino acids: without any virulent residue	≤61 amino acid	Total[β]
2017	L					R	R	L	45	93	2	0	19	112
						R			19					
	L					R		L	12					
	L		I			R	R	L	11					
						R	R	L	4					
	L	S				R	R	L	2					

	L				R	R	L	22					
	L		I		R	R	L	15					
**2016**					R	R	L	10	51	1	0	9	60
	L					R	L	2					
	L	S		L			L	1					
					R			1					
	L				R	R	L	92					
	L				R		L	3					
**2015**					R			3	101	0	0	17	118
	L					R	L	2					
	L		I		R		L	1					
	L				R	R	L	121					
	L		I		R	R	L	77					
**2014**	L				R	R		2	205	3	0	25	230
	L	S			R	R	L	3					
	L				R		L	1					
	L					R	L	1					
	L				R	R	L	84					
	L		I		R	R	L	12					
**2013**	L				R			3	101	0	0	13	115
	L					R	L	2					
	L					R		1					
**2012**	L	S			R	R	L	1	1	1	0	0	1
**2011-05**									No isolate				
**2004**	L	S			R	R	L	1	1	1	0	0	1
						R		3					
**2003**	L					R		2	6	1	0	0	6
	L	S			R	R	L	1					
**Total**								**560**	**560**	**9**	**0**	**83**	**643**

SUPPLEMENTARY TABLE 1 Frequency of Virulent PB1-F2 Residues in Human Influenza A Virus Isolates. Highlighted in: yellow – the residues enhancing the cytotoxicity of A/Puerto Rico/8/1934(H1N1) PB1-F2,[31] turquoise – the residues enhancing inflammatory properties of pandemic H3N2 A/Hong Kong/1/1968(H3N2) PB1-F2,[16] and pink – the reside linked to the higher virulence of pandemic 1918(H1N1) and HPAIV(H5N1).[12,13] The annotation of swine origin viruses in H3N2 dataset is based on the data from the Centers for Disease Control and Prevention (Atlanta, GA).

[α]Cytotoxic (I68, L69, and V70)[31] and inflammatory (L62, R75, R79, and L82)[16] residues (according to PB1-F2 amino acid numbering).

[β]The S66 was not included in the total count when combined with other virulent residues.

[a]Eurasian avian-like lineage

[b]North American triple reassortant lineage

[c]Reassortant strains with human seasonal HA and NA

[d]European lineage

[e]North American lineage

Note, the number of human cases infected by IAVs is higher than counts included in our analyses because of the unavailability of complete PB1-F2 ORF sequences in public databases. Reassortment in swine viruses is very common and complex than human strains and out of the scope of this study.
